# Association between perceived stress and the risk of continued opioid use after total hip arthroplasty in patients with osteoarthritis: a Danish registry-based study of 1,727 individuals

**DOI:** 10.2340/17453674.2025.44759

**Published:** 2025-10-03

**Authors:** Nina M EDWARDS, Heidi A R JENSEN, Alma B PEDERSEN

**Affiliations:** 1 Department of Orthopaedic Surgery, Regionshospitalet Horsens; 2 Department of Orthopaedic Surgery, Aarhus University Hospital; 3 National Institute of Public Health, University of Southern Denmark, Copenhagen; 4 Department of Clinical Epidemiology, Department of Clinical Medicine, Aarhus University and Aarhus University Hospital, Aarhus, Denmark

## Abstract

**Background and purpose:**

Continued opioid use persists in up to one-third of patients 12 months after total hip arthroplasty (THA). Psychological factors, including stress, may influence pain and therefore opioid consumption, yet the effect of stress history on opioid use after THA remains unclear. We aimed to examine the association between perceived stress and the risk of continued opioid use following THA in patients with osteoarthritis.

**Methods:**

Based on data from the Danish National Health Surveys in 2013 and 2017, a total of 1,727 individuals completed the Perceived Stress Scale and later underwent THA, tracked through the Danish Hip Arthroplasty Registry. All were over the age of 35. Patients were classified by stress level (high vs low stress). Continued opioid use was defined as ≥ 2 opioid prescriptions 1–12 months post-surgery, recorded in the Danish National Prescription Database. Adjusted prevalence differences and adjusted prevalence ratios were calculated using log-binomial regression, controlling for sex, age, comorbidities, and education.

**Results:**

Of 258 patients with high stress level, 68 (26%) had continued opioid use, compared with 224 (15%) of the 1,469 patients with a low level. We showed higher ratios in high stress patients (adjusted prevalence difference 9.2; 95% confidence interval [CI] 3.6–14.8, adjusted prevalence ratio 1.5 [CI 1.2–1.9]). Median morphine milligram equivalents (MME) were higher for high stress with a median difference of 1,230 (interquartile range 1,025–3,745).

**Conclusion:**

High levels of perceived stress before THA are associated with a higher risk of continued opioid use and greater opioid consumption in the first postoperative year. These findings suggest the potential for preoperative stress screening and targeted interventions to reduce postoperative opioid use.

Total hip arthroplasty (THA) is a highly effective surgical intervention for patients with osteoarthritis, a degenerative joint disease that causes pain and impaired mobility [1]. The primary objective of THA is to alleviate pain and restore joint function, thereby improving patients’ quality of life. Ideally, this reduction in pain is reflected in decreased reliance on analgesic medications, particularly opioids, which are commonly prescribed in the postoperative period [2,3]. However, despite the overall success of this surgical procedure, the postoperative management of pain remains a significant challenge, and continued opioid use is seen in 4–29 % of patients 12 months after THA [4]. This is concerning, as opioid use is associated with a range of adverse side effects [5,6].

Psychological factors, including perceived stress, have been shown to influence pain perception, recovery outcomes, and opioid consumption [6,7]. Stress is a complex response to environmental, social, and personal factors, and has been linked to both the initiation and continuation of opioid use [7-9]. However, inconsistency in the definition of stress is an important barrier to evaluate stress as a potential risk factor. Further, it is not evident whether these definitions are perceived as stressful by the individual [7]. The Perceived Stress Scale (PSS), a validated tool designed to measure the extent of unpredictability, uncontrollability, and overload an individual perceives, may provide a more accurate measure of perceived stress [7,10-12].

While stress has been associated with opioid use in other settings, no study has specifically quantified the differences in opioid consumption between THA patients reporting high vs low levels of perceived stress before surgery. This study aims to address this gap by examining the association between self-reported perceived stress and the risk of continued opioid use 12 months after THA in osteoarthritis patients.

## Methods

### Study design

Our study was conducted as a population-based cohort study in Denmark based on several nationwide registers. This study was reported following the Strengthening the Reporting of Observational Studies in Epidemiology (STROBE) statement and the REporting of studies Conducted using Observational Routinely-collected Data [13] statement.

### Setting

All citizens are guaranteed free tax-supported medical care provided by the Danish Health Service. All citizens are assigned a unique civil registration number at birth or upon immigration, which is included in all Danish registries allowing unambiguous individual-level record linkage, e.g., to complete long-term follow-up of all Danish inhabitants in Denmark.

We used the Danish National Health Survey (DNHS), which is a large nationwide health survey among the general population aged 16 years or older conducted by 5 Danish regions, the Danish Health Authority, and the National Institute of Public Health at the University of Southern Denmark [14]. The survey has been conducted regularly since 2010, and data was collected by self-administered paper-and-pencil or web-based questionnaires. The questionnaire in each survey wave contained approximately 100 questions, e.g., on quality of life, health behavior, and stress, and validated questionnaire scales were included such as the PSS [10]. Data from the 2013 and 2017 survey were used in this study.

We used the following registries:

1. The Danish Civil Registration System, which holds data on vital status, migrations, age, and sex.
2. The Danish Hip Arthroplasty Registry, which holds information on—but not restricted to—primary THA surgeries, primary diagnosis, and date of surgery. All patients over the age of 35 undergoing primary THA in Denmark from January 1, 1995 to December 31, 2018 with the primary diagnosis of idiopathic osteoarthrosis (completeness of DHR: 91%–98%) were identified.
3. The Danish National Patient Registry, which contains discharge dates and diagnoses from all hospitalizations since 1977, and outpatient clinic and emergency room contacts since 1995. We collected data for each patient on comorbidity history. We measured the comorbidity status by Charlson Comorbidity Index (CCI) score. Patients were classified in 1 of the 3 levels of the CCI score: low (a score of 0); medium (a score of 1–2); and high (a score of 3 or more).
4. The Population Education Registry, which obtains information on highest obtained educational level. Data is generated from administrative records of educational institutions. Educational level was categorized into low (elementary school), medium (more than elementary school but less than university degree), and high (university degree).
5. The Danish National Prescription Registry, which holds data on all prescriptions for reimbursed drugs dispensed by community pharmacies in Denmark since 2004. For each THA patient, we retrieved information on any dispensed opioid prescription 1 year after THA surgery.

### Study population

We identified all individuals, who completed all items on the Perceived Stress Scale (PSS) [14]. All were aged 16 years or older and resident in Denmark. Individuals who, after answering the PSS, underwent first-time THA due to osteoarthritis as recorded in the DHR, were included. We only included the first THA during 1995–2017 in the study population to avoid dependency of observations. Only patients with full follow-up 1–12 months after surgery and had a fully observable outcome were included. Patients who either died or emigrated (less than 5 individuals), were excluded from analyses.

### Perceived Stress Scale

The PSS was included in the questionnaire, which is a global stress measure scale developed to assess the extent of unpredictability, uncontrollability, and overload perceived by individuals [10]. The PSS also measures the extent to which external demands seem to exceed the individual’s perceived ability to cope [15]. The scale consists of 10 questions regarding the frequency to which individuals have, within the previous month, had certain feelings and thoughts using a 5-point scale (0 = never, 1 = almost never, 2 = sometimes, 3 = fairly often, 4 = very often) [10,16]. The PSS has been validated, and the Danish consensus version is considered feasible for use in clinical research settings [10,15]. To gain power for the statistical analyses, the PSS was dichotomized. We have used different cutoff points for women and men because stress level measured with the PSS was shown to be higher for women than men [17]. As the PSS does not have score cutoffs, the cutoff points were found by categorizing each sex population according to percentiles of stress scores and classifying participants in a high stress level by taking the 20% percentile of the participants experiencing the highest levels of stress [18]. This method of finding cutoff points was described and used previously [19]. Individuals were subsequently categorized within high levels of stress, if they had a score ≥ 17 for women and ≥ 15 for men or within a low level of stress if they had a score < 17 for women and < 15 for men. Individuals reporting no stress were included in the group of low level of stress [16].

### Outcome

The primary outcome was continued opioid use defined as ≥ 2 opioids dispensing occasions in the 1–12 months following THA. As currently there is no standard definition and consensus of chronic opioid use, the definition of chronic use in our study was guided by recent reviews and clinical expertise [20]. We excluded the first 30 days, as opioid use postoperatively in close proximity to the surgery is anticipated. Information on opioids was collected through ATC codes. The secondary outcome was milligram morphine equivalent (MME) doses calculated using a conversion factor corresponding to the specific opioid types (Supplementary Table 1) [21].

### Statistics

Baseline characteristics of the population were described using counts and percentages. Baseline balance was assessed using standardized differences. We calculated period prevalences of opioid use by level of perceived stress. In the main analysis we calculated the crude prevalence differences and the prevalence ratios with 95% confidence intervals (CI) using log-binomial regression. Prevalence differences and prevalence ratios were further adjusted for sex, age, comorbidities, and education providing adjusted prevalence differences and adjusted prevalence ratios. We additionally adjusted for preoperative opioid use. Analyses were performed overall and by preoperative opioid use defined as ≥ 1 opioid dispensed 0–6 months before THA.

The MME was calculated 1–12 months after THA for both groups and presented in bar plots. The confidence interval for the difference in medians was estimated using a bootstrap procedure.

The mean number of days between completing the survey and THA surgery was calculated and presented in tables.

2 sensitivity analyses were performed to assess the robustness of the findings by varying the definition of opioid use. The first sensitivity analysis was performed by changing the definition of opioid use after THA from 1–12 months to 3–12 months with ≥ 2 opioids dispensing occasions. The second sensitivity analysis was performed by changing the outcome from ≥ 2 opioids dispensing occasions 1–12 months after THA to ≥ 2 opioids dispensing occasions in 2 different quarters 3–12 months after THA. Quarters were defined as 4th–6th, 7th–9th, and 10th–12th months after THA.

The assumption was made that there were no systematic differences between missing values and the observed values, i.e., data are missing completely at random [22].

The statistical analyses were performed in STATA version 16 (StataCorp LLC, College Station, TX, USA) and R version 4.1.0 (R Foundation for Statistical Computing, Vienna, Austria).

### Ethics, use of AI tools, funding, and disclosures

The study was approved by the Danish Data Protection Agency (journal number 2015-57-0002) and Aarhus University (journal number 2016-051-000001). AI tools were not used. No funders have supported this study. The authors report no conflicts of interest. Complete disclosure of interest forms according to ICMJE are available on the article page, doi: 10.2340/17453674.2025.44759

## Results

**Figure 1:**
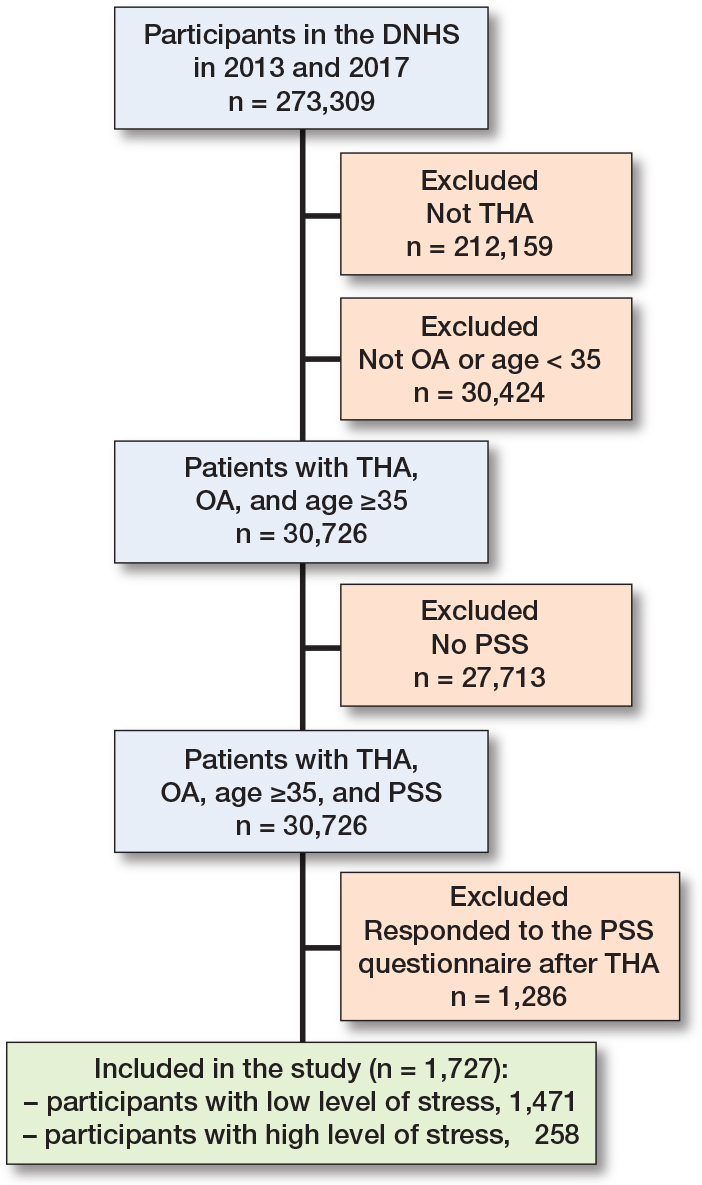
Patient flowchart.

We identified 244,645 individuals (162,283 individuals in 2013, 54% of those invited, and 183,372 in 2017, 59% of those invited), who completed all items on the Perceived Stress Scale (PSS) [14]. We excluded individuals who were younger than 35 years of age (n = 30,726) to correspond with the standard THA population. Among the remaining individuals, 1,727 individuals were included (Figure 1). Among these, 258 (15%) reported a high level of perceived stress, while 1,469 (85%) reported low stress prior to THA. Despite similarities in sex and age distribution, differences were observed in educational level and CCI distribution between the 2 groups. Patients with high levels of perceived stress had a higher proportion of individuals with low education (42%) than patients with low stress (28%). Similarly, patients with a high level of perceived stress had a higher proportion of individuals with high CCI (12%) than patients with low stress (6%) (Table 1).

**Table 1:** Patient characteristics. Values are count (%) unless otherwise specified

Characteristics	Low level of stress ^a^	High level of stress ^a^	Standardized differences
Sex			
Female	794 (54)	161 (62)	0.16
Male	677 (46)	97 (38)	0.16
Age, median	71	72	
Age group			
< 60	193 (13)	40 (15)	0.06
60–69	503 (34)	67 (26)	0.18
> 70	773 (53)	151 (59)	0.12
Charlson Comorbidity Index **^b^**			
Low	1,029 (70)	145 (56)	0.29
Medium	353 (24)	83 (32)	0.18
High	87 (6)	30 (12)	0.21
Education **^c^**			
Low	410 (28)	109 (42)	0.30
Medium	681 (46)	112 (44)	0.04
High	378 (26)	37 (14)	0.30

a Measured with self-reported Perceived Stress Scale.

b Charlson Comorbidity Index (CCI): patients were classified on 1 of the 3 levels of the CCI score: low (0); medium (1–2); and high (≥ 3 ).

c Educational level was categorized into low (elementary school), medium (more than elementary school but less than university degree), and high (university degree).

### Opioid consumption and dosage

Notably, 26% of patients with high levels of perceived stress continued opioid use after THA, compared with 15% of patients with low stress (Table 2).

**Table 2:** Opioid use 1–12 months after total hip arthroplasty (THA) by Perceived Stress Scale based on ≥ 2 opioid dispensing occasions

	Overall		Non-users ^a^		Users ^b^	
	Low level of stress n = 1,471	High level of stress n = 258	Low level of stress n = 1,019	High level of stress n = 144	Low level of stress n = 450	High level of stress n = 114
Outcome, n (%)	224 (15)	68 (26)	78 (7.7)	19 (13)	148 (33)	53 (46)
Crude PD (CI)	1	12.5 (6.7–18.3)	1	5.5 (–2.0 to 8.3)	1	13.6 (3.5–23.7)
aPD (CI)	1	9.2 (3.6–14.8)	1	3.1 (–2.0 to 8.3)	1	10.7 (0.5–20.8)
Crude PR (CI)	1	1.8 (1.4–2.3)	1	1.7 (1.1 to 2.8)	1	1.4 (1.1–1.8)
aPR (CI)	1	1.5 (1.2–1.9)	1	1.4 (0.9 to 1.6)	1	1.2 (1.0–1.6)
aPR (CI) **^c^**	1	1.4 (1.1–1.8)				

CI: 95% confidence interval, PD: prevalence difference, PR: prevalence ratio, aPD and aPR: adjusted for sex, age, comorbidity measured with Charlson Comorbidity Index, and education. Based on ≥ 2 opioid dispensing occasions 1–12 months after THA.

a Non-users: defined as no opioid dispensing 0–6 months before THA.

b User: defined as ≥ 1 opioid dispensed 0-–6 months before THA.

c Additionally adjusted for preoperative opioid use

The adjusted prevalence difference of continued opioid use between high and low levels of perceived stress was 9.2 (CI 3.6–14.8). The adjusted prevalence ratio for continued opioid use was 1.5 (CI 1.2–1.9) (see Table 2). When additionally adjusting for preoperative opioid use, the adjusted prevalence ratio declined to 1.4 (1 CI.1–1.8).

When defining the study population by preoperative opioid use, the adjusted prevalence difference was 3.1 (CI –2.0–8.3) and the adjusted prevalence ratio was 1.4 (CI 0.9–2.3) for non-users, and the adjusted prevalence difference was 10.7 (CI 0.5–20.8) and the adjusted prevalence ratio was 1.2 (CI 1.0–1.6) for users (see Table 2).

**Figure 2:**
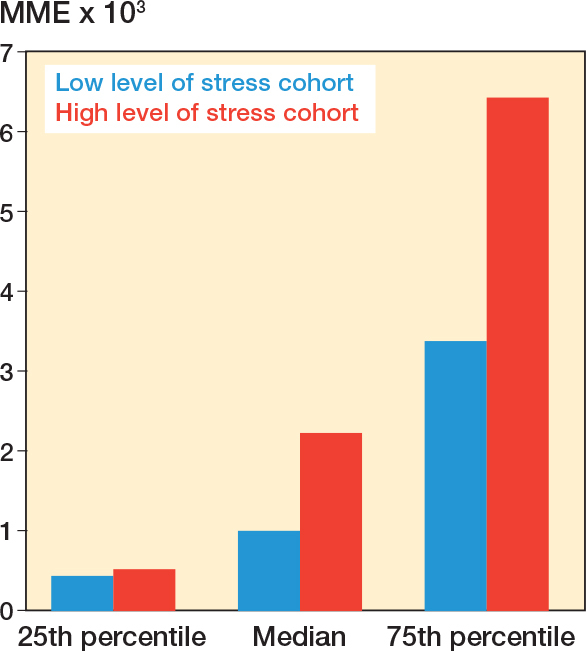
Milligram morphine equivalent (MME) dose by Perceived Stress Scale.

The median MME dose was 2,230 for patients who reported high levels of perceived stress (Figure 2). The MME was 1,000 for patients who reported low levels of stress. The median difference was 1,230 mg morphine (interquartile range 1,025–3,745).

The mean number of days between completing the survey and THA surgery was 825 for patients with low levels of perceived stress, whereas the mean number of days was 258 for patients with high levels of perceived stress (Table 3).

**Table 3:** Median number of days between completing the survey and surgery by Perceived Stress Scale

	Low level of stress	High level of stress	Median difference
Median number of days	736	629	107
25% percentile	304	251	657
75% percentile	1,320	1,326	1,153

### Sensitivity analyses

When defining opioid use as ≥ 2 opioids dispensing occasions 3–12 months after THA, the adjusted prevalence difference was 9.8 (CI 4.4–15.3) and the adjusted prevalence ratio was 1.6 (CI 1.3–2.1) (Supplementary Table 2). When defining opioid use as ≥ 2 opioids dispensing occasions 3–12 months after THA in 2 different quarters, the adjusted prevalence difference was 8.6 (CI 3.6–13.6) and the adjusted prevalence ratio was 1.9 (CI 1.4–2.5) (Supplementary Table 3).

## Discussion

We aimed to examine the association between perceived stress and the risk of continued opioid use following THA in osteoarthritis patients. We found a significant association between perceived stress and increased risk of continued opioid use after THA and greater MME dose.

While stress has been associated with opioid use in other settings, our study has further quantified the differences in opioid consumption between THA patients reporting high vs low levels of perceived stress before surgery [9]. We found that the median MME dose was 2,230 for patients who reported high levels of perceived stress and 1,000 for patients who reported low levels. This difference has not been quantified in a similar setting before.

Differences in the definition of continued opioid use are seen in the literature [23]. We defined continued use as the usage of opioids beyond 30 days. However, the definition of continued use as the usage of opioids beyond 90 days would be in line with the International Association for the Study of Pain. They define chronic postoperative pain as pain that develops after a surgical procedure and persists for at least 3 months after surgery [24,25]. We therefore performed the sensitivity analyses changing the time period for the usage of opioids from 30 to 90 days. This showed no considerable changes in our results, thus contributing to the evidence of robustness to our conclusions. Further, using ≥ 2 filled prescriptions to define opioid use is a more adequate definition than using 1 filled prescription in a specified time window, as these definitions fail to identify consistent use [23].

The number of opioid dispensing occasions and the time in which they are dispensed could also influence our results. For example, an opioid dispensed twice near the end of follow-up would be misclassified as continued use, but in fact be due to a restart of opioid use for conditions other than THA. As our information is based solely on registry data, we have no information regarding the indication for which the opioid has been prescribed. Therefore, we performed sensitivity analyses to test the robustness of our results, changing the definition of opioid use after THA. Here we found similar results showing that again the difference in the adjusted prevalence ratio of opioid use after THA is influenced not by opioid use but by the individual’s perception of stress.

### Stress and the risk of continued opioid use

We observed similar adjusted prevalence difference and adjusted prevalence ratio among preoperative opioid users and non-users; however, with a smaller magnitude among opioid users. This suggests that associations were not driven by opioid use in general, which is often assumed, but rather by the individual him/herself. This is further established when controlling for preoperative opioid use, showing that high stress is still a significant predictor independent of prior opioid use.

The association between stress and continued opioid use may be attributed to several factors. High stress levels could exacerbate the perception of pain, leading to greater opioid consumption for pain relief [26]. Additionally, stress is known to affect psychological and physiological responses, potentially influencing pain management [27].

The perception of stress is influenced by the perception of control or mastery to sustain confidence in the ability to cope with stressful life events [7,11]. Further, an individual’s perception of stress may influence the use of information sources. This could result in finding it difficult to pursue and utilize information and to implement the appropriate protective measures against health risks [28]. Additionally, stress could be related to postoperative complications. The difficulties in adopting these protective measures may prevent the acknowledgment of early onsite symptoms and thereby prevent adequate precautions. The higher proportion of low educational levels among the patients with high perceived stress suggest that educational attainment might also play a role in stress perception and pain management. While we adjusted for education, residual confounding could explain some of our findings as lower education levels could be associated with reduced health literacy, making it more challenging for patients to understand and manage their pain effectively without reliance on opioids [29]. There is substantial evidence that higher education is positively correlated with health, and that this is driven by the cognitive ability to process complex information regarding healthy behavior, psychosocial resources, and health literacy (Supplementary Figure 1) [30,31].

### Strengths

First, this study is based on nationwide registry data where information on opioid use, CCI, and education was collected on an individual patient level enabling detailed definition of these variables and adjustment for confounding.

Furthermore, evaluating stress as an exposure using self-reported levels of stress is also a strength. Self-report directly measures what the individual feels, which is the exposure of interest. However, using self-reported measurements can also be a weakness, as it is measured only once, and may not reflect fluctuations. Further, in observational studies, individuals with more pain or opioid dependence might report higher stress, complicating causal inference. Defining stress solely as the presence of psychological diagnoses or a series of potentially stressful life events is incomplete, as such events may not necessarily be experienced as stressful by the individual [7]. Conversely, minor events or isolated diagnoses that are not typically classified as stressful may still be perceived as highly stressful. Perception of stress is strongly influenced by an individual’s sense of control and confidence in their ability to cope with life events. Whereas specific psychiatric conditions such as depression and anxiety have been extensively studied, perceived stress offers a broader assessment of psychosocial strain, encompassing overall mental state and coping capacity [8]. This is captured in the PSS, which is validated and, further, the Danish consensus version is rated as feasible for use in clinical research settings, which also is a strength [10,15].

### Limitations

First, our study had a response rate of 54–59% [14]. The response rate was particularly low among older women aged > 85, whereas the typical THA patient is a women (57%) over the age of 70 (53%) [14,32]. Recently, we showed that participants in the surveys had similar age and sex distribution but appeared to be slightly healthier when measured with comorbidity burden, medication, and healthcare utilization than non-participants who have subsequently undergone THA [33]. Thus, our absolute risks of opioid use might underestimate the true risks because healthy patients take fewer opioids and the generalizability of our results applies to healthier THA patients. Further, the response rate was low among individuals with < 10 years of education [14]. We saw a higher proportion of low education among the patients with high perceived stress and low education in association with higher opioid use after THA, potentially affecting our relative risk estimates [34]. To reduce differential responder bias, surveys using a weighting method could be used [35]. We were, however, not able to apply this method because data on non-responders was not available for us in this study.

The timeline of 2 years between completing the survey and surgery leads to potential misclassification of the true preoperative stress level. Stress could increase as one approaches surgery or change due to intervening events, biasing our association towards the null. However, perception of stress exposure and of coping resources, as assessed by the PSS, is a relatively stable characteristic that varies only slightly over short intervals and only moderately over longer intervals. The determination of a large between-person component of variance suggests that PSS scores are influenced, to a large extent, by factors that are relatively stable [36]. These factors could be an individual’s personality or other characteristics that we think of as more trait-like, in that they exhibit little fluctuation across many years, or even across a person’s lifetime.

This study clarifies the complex relationship between perceived stress and postoperative opioid use, highlighting the importance of integrating psychological assessment into pain management for patients undergoing total hip arthroplasty. Comprehensive strategies, including stress management, patient education, and tailored pre- and postoperative counseling, may help reduce the risk of prolonged opioid use, particularly among patients with high perceived stress or lower educational attainment.

### Conclusion

Perceived stress increases the risk of continued opioid use and MME dose after THA in osteoarthritis patients.

*In perspective,* regular follow-up to monitor pain, rehabilitation progress, and opioid consumption could further support early identification and intervention. Addressing stress and incorporating patient education may reduce opioid dependence and improve postoperative outcomes. Further research is warranted to explore targeted preoperative, intraoperative, and postoperative interventions for stress management in this patient population.

### Supplementary data

Supplementary Tables 1–3 and Supplementary Figure 1 are available as supplementary data on the article page, doi: 10.2340/17453674.2025.44759

## Supplementary Material



## Notes

NME drafted the manuscript. NME and AP conceived and designed the study, NME, HARJ, and AP interpreted the results and revised the manuscript.

Handling co-editors: Keijo Mäkelä and Robin Christensen

*Acta* thanks Michael Axenhus for help with peer review of this manuscript.
