# Supplementary material for: Association between perceived stress and the risk of continued opioid use after total hip arthroplasty in patients with osteoarthritis: a Danish registry-based study of 1,727 individuals
Source: Acta Orthop. 2025 Oct 3;96:740–6. doi: 10.2340/17453674.2025.44759 (PMC12492309; doi:10.2340/17453674.2025.44759)
Supplement: Supplementary file 1 [file ActaO-96-44759-s1.pdf]

Supplementary Table 1. Morphine milligram equivalent conversion factors

**Opioid**	Conversion factor
**Oral medication**	
Morphine	1
Hydromorphone	5.0
Oxycodone	1.5
Oxycodone + naloxone	1.5
Tramadol	0.2
Tapentadol	0.4
Methadone	4.7
Codeine	0.1
**Parenteral medication**	
Morphine	3.0
Oxycodone	3.0
Pethidine	0.4
Buprenorphine	75.0
Methadone	13.5
**Sublingual medication**	
Fentanyl	100.0
Buprenorphine	38.8
**Transdermal medication**	
Fentanyl	112.5
Buprenorphine	91.7

Supplementary Table 2. Sensitivity analysis: opioid use 3–12 months after THA by Perceived Stress Scale based on ≥ 2 opioid dispensing occasions

	Perceived stress scale		
**Exposure**	**Low level of stress**	**High level of stress**	**Difference**
**Patients**	**1,469**	**258**	**1,211**
**Outcome, n (%)**	**188 (13)**	**66 (26)**	**122**
**Crude RD (CI)**	**1**	**12.8 (7.2–18.4)**	
**aRD (CI)**	**1**	**9.8 (4.4–15.3)**	
**Crude PR (CI)**	**1**	**2.0 (1.6–2.6)**	
**aPR (CI)**	**1**	**1.6 (1.3–2.1)**	

n: number, CI: 95% confidence interval, RD: prevalence difference, PR: prevalence ratio, aRD and aPR: adjusted for sex, age, comorbidity measured with Charlson Comorbidity Index, and education.

Supplementary Table 3. Sensitivity analysis: opioid use 3–12 months after THA by Perceived Stress Scale based on ≥ 2 opioid dispensing occasions in 2 different quarters

	Perceived stress scale		
**Exposure**	**Low level of stress**	**High level of stress**	**Difference**
**Patients, n**	**1,469**	**258**	**1,211**
**Outcome, n (%)**	**136 (9.3)**	**53 (21)**	**83**
**Crude RD (CI)**	**1**	**11.8 (6.1–16.4)**	
**aRD (CI)**	**1**	**8.6 (3.6–13.6)**	
**Crude PR (CI)**	**1**	**2.2 (1.6–3.0)**	
**aPR (CI)**	**1**	**1.9 (1.4–2.5)**	

For abbreviations, see Supplementary Table 2

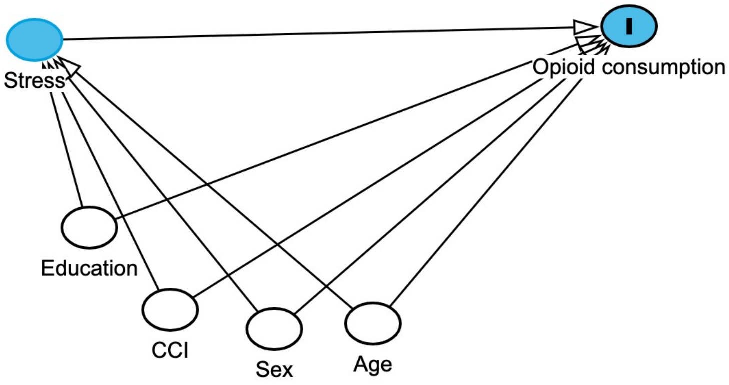

Supplementary Figure 1. Directed acyclic graph (DAG).
